# Prioritizing zoonotic diseases using a multisectoral, One Health approach for The Economic Community of West African States (ECOWAS)

**DOI:** 10.1186/s42522-021-00055-6

**Published:** 2021-11-23

**Authors:** Grace W. Goryoka, Virgil Kuassi Lokossou, Kate Varela, Nadia Oussayef, Bernard Kofi, Vivian Iwar, Casey Barton Behravesh

**Affiliations:** 1 Centers for Disease Control and Prevention (CDC) , Atlanta, USA; 2 ECOWAS Regional Center for Surveillance and Disease Control, Abuja, Nigeria; 3 West African Health Organization, Bobo-Dioulasso, Burkina Faso; 4 ECOWAS Directorate of Environment and Natural Resources, Abuja, Nigeria; 5 ECOWAS Regional Animal Health Center, Bamako, Mali

**Keywords:** Zoonotic diseases, Disease prioritization, One Health, Multisectoral collaboration, One Health Coordination Mechanisms, Infectious disease outbreak, Preparedness and response, Prevention and control, RCSDC, RAHC, West Africa

## Abstract

**Background:**

Zoonotic diseases pose a significant threat to human, animal, and environmental health. The Economic Community of West African States (ECOWAS) has endured a significant burden of zoonotic disease impacts. To address zoonotic disease threats in ECOWAS, a One Health Zoonotic Disease Prioritization (OHZDP) was conducted over five days in December 2018 to prioritize zoonotic diseases of greatest regional concern and develop next steps for addressing these priority zoonoses through a regional, multisectoral, One Health approach.

**Methods:**

The OHZDP Process uses a mixed methods prioritization process developed by the United States Centers for Disease Control and Prevention. During the OHZDP workshop, representatives from human, animal, and environmental health ministries from all 15 ECOWAS Member States used a transparent and equal process to prioritize endemic and emerging zoonotic diseases of greatest regional concern that should be jointly addressed by One Health ministries and other partners. After the priority zoonotic diseases were identified, participants discussed recommendations and further regional actions to address the priority zoonoses and advance One Health in the region.

**Results:**

ECOWAS Member States agreed upon a list of seven priority zoonotic diseases for the region – Anthrax, Rabies, Ebola and other viral hemorrhagic fevers (for example, Marburg fever, Lassa fever, Rift Valley fever, Crimean-Congo Hemorrhagic fever), zoonotic influenzas, zoonotic tuberculosis, Trypanosomiasis, and Yellow fever. Participants developed recommendations and further regional actions that could be taken, using a One Health approach to address the priority zoonotic diseases in thematic areas including One Health collaboration and coordination, surveillance and laboratory, response and preparedness, prevention and control, workforce development, and research.

**Conclusions:**

ECOWAS was the first region to use the OHZDP Process to prioritize zoonotic disease of greatest concern. With identified priority zoonotic diseases for the region, ECOWAS Member States can collaborate more effectively to address zoonotic diseases threats across the region using a One Health approach. Strengthening national and regional level multisectoral, One Health Coordination Mechanisms will allow ECOWAS Member States to advance One Health and have the biggest impact on improving health outcomes for both people and animals living in a shared environment.

## Background

Preventing and controlling zoonotic diseases requires a multisectoral, One Health approach. Zoonotic diseases are diseases spread between animals and people. Most known human infectious diseases and about three-quarters of newly emerging infections originate from animals [1]. Zoonotic diseases pose a significant threat at the human-animal-environment interface, and have economic and social impacts. The need to involve multiple sectors in zoonotic disease events has historically been a challenge in addressing threats at the human-animal-environment interface [2–4], and highlights the critical need for a multisectoral, One Health approach to address these emerging health threats. The U.S. Centers for Disease Control and Prevention (CDC) developed a One Health Zoonotic Disease Prioritization (OHZDP) Process that aims to overcome these challenges to address zoonotic disease threats by identifying collaborative priorities and strengthening multisectoral collaboration in over 25 subnational, national, and regional levels [5–7].

The Economic Community of West African States (ECOWAS), made up of 15 Member States – namely Benin, Burkina Faso, Cape Verde, Côte d’Ivoire, The Gambia, Ghana, Guinea, Guinea Bissau, Liberia, Mali, Niger, Nigeria, Sierra Leone, Senegal, and Togo—has endured a significant burden of zoonotic disease impacts. ECOWAS Commission was established in 2006 through a revised treaty to strengthen the operations of ECOWAS institutions and agencies [8]. ECOWAS is an economic and integration community aimed at fostering relations among its Member States, promoting free movement of people and goods, maintaining and enhancing economic growth, raising the standard of living, and contributing to the progress and development of the African continent [9]. ECOWAS has the vision of a peaceful, borderless, and integrated region where citizens enjoy free movement and access to shared resources in accordance with the principles of democracy, the rule of law, multisectoral collaboration, and good governance [9]. The free movement of people throughout the region, may allow diseases to move just as freely. The health of humans, animals, and the environment are fundamental to the livelihood and productivity of all people, livestock, and wildlife. To foster a One Health approach in West Africa, ECOWAS Commission established a Department of Agriculture, Environment & Water Resources and the West African Health Organization (WAHO) which comprise of different Directorates (Environment & Natural Resources Directorate, Regional Center for Surveillance and Disease Control, and the Regional Animal Health Center). These Directorates work closely to foster One Health in the region through the implementation of the Libreville Declaration on Health and Environment in Africa [10] and the Luanda Commitment [11] under the United Nations Environment Programme (UNEP) and the World Health Organization (WHO) auspices.

Major infectious disease outbreaks have seriously threatened the stability of the region [12–14], thus it is important to coordinate prevention and control activities across all ECOWAS Member States.

Concerned about the 2014–2016 Ebola Virus Disease outbreak in West Africaand in line with the Resolution A/RES/70/1 of the General Assembly of the United Nations that adopted the 2030 Agenda for Sustainable Development Goals (SDGs) and targets,West African governments committed themselves in the Dakar Consultation to establishing robust national and regional multisectoral,, One Health Coordination Mechanisms. A multisectoral, One Health coordination mechanism is “any formalized, standing, group that acts to strengthen or develop collaboration, communication, and coordination across the sectors responsible for addressing zoonotic diseases and other health concerns at the human-animal-environment interface” [15]. During the Dakar consultation, the West African governments identified clear roles and responsibilities and accountability of relevant stakeholders for their multisectoral, One Health coordination mechanism [16]. Countries also promised to better integrate One Health into national and regional multisectoral plans for infectious disease prevention and control, environmental protection, resilience, and food security. Examples of efforts to integrate One Health include creating a multisectoral, One Health Coordination Mechanism [17–19], establishing a One Health secretariat with clear governance structure including all sectors, developing and implementing a One Health action plan [20], promoting collaboration mechanisms across sectors to prevent and detect zoonotic diseases, and establishing technical working groups or taskforces to monitor the progress made towards One Health priorities.

After Dakar’s meeting, ECOWAS countries also validated and adopted in Abuja in July 2017 a regional strategic One Health Framework in order to foster a shared vision in the implementation of One Health agenda to further strengthen prevention, detection, and response to infectious disease threats, including zoonoses and antimicrobial resistance [21].

To address zoonotic disease challenges within the ECOWAS region, an OHZDP Workshop was conducted in December 2018, the first regional workshop of its kind. The purpose of this workshop was to use a multisectoral, One Health approach to prioritize zoonotic diseases of greatest regional concern for ECOWAS. This publication describes the OHZDP Process and outcomes of the ECOWAS OHZDP Workshop and next steps needed to advance health security in West Africa and beyond. ECOWAS was the first region in the world to conduct an OHZDP workshop and serves as an example for other regions interested in strengthening One Health collaborative efforts at a regional level.

## Methods

The OHZDP Process uses a mixed methods approach developed by the U.S. Centers for Disease Control and Prevention (CDC) One Health Office. The original methods of the OHZDP Process have been previously described in detail [5, 6]. In short, the OHZDP Process uses established qualitative, semi-quantitative, and quantitative methods to prioritize zoonotic diseases of greatest concern and develop recommendations for the priorities using equal input from One Health sectors through a transparent, collaborative process [5, 6].

### Before the workshop

Workshop organizers began the technical and logistical preparations for this prioritization in June 2018. A core planning team (CPT) was established to prepare and plan for the workshop and consisted of members from CDC’s One Health Office, ECOWAS, the Food and Agriculture Organization of the United Nations (FAO), United States Agency for International Development (USAID), and the Republic of Senegal, the host country.

The specific goals of the OHZDP in ECOWAS were 1) to use a multisectoral, One Health approach to prioritize endemic and emerging zoonotic diseases of greatest regional concern that should be jointly addressed by human, animal, and environmental health ministries and other partners using a One Health approach, and 2) to develop next steps and action plans for addressing the prioritized zoonotic diseases through a multisectoral, One Health approach.

The CPT was responsible for all technical and logistical preparations for the workshop and began preparations six months in advance. The CPT developed an initial region-specific zoonotic disease list for prioritization by reviewing and incorporating zoonotic diseases from the human and animal reportable disease lists from all 15 Member States. A disease was added to the list if it was known to spread between animals and people and thought to occur in or surrounding the ECOWAS region. Additional zoonoses were added through a literature search through PubMed and Google Scholar that identified zoonotic diseases present within ECOWAS and bordering countries in the past 10 years. Keywords used in the literature review to identify zoonotic diseases present within the ECOWAS region included each country’s name within the ECOWAS region and the following terms: “zoonoses”, “zoonotic disease”, “prevalence”, “incidence”, “outbreak”. CPT members and ECOWAS reviewed this initial list to ensure zoonotic diseases of greatest regional importance were included, prior to sharing with workshops participants.

Preparations for this regional workshop included participant identification, including Member States, observers, and facilitators. ECOWAS sent invitations to each Member State to request one representative from their human, animal, and environmental health ministries, three total representatives per Member State. The Member States’ representatives held a technical leadership role within their ministry for zoonotic disease work, provided key input and decision-making, and were willing and able to carry the prioritization process forward. Observers, invited by ECOWAS, were identified to help support post-workshop collaborative activities and participated in select open discussions during the workshop (Table 1). Trained and experienced CDC and regional FAO facilitators were selected by ECOWAS to facilitate this regional OHZDP Workshop. Additionally, ECOWAS also supported logistics and travel for Member States.

**Table 1:** Participating organizations and their role for the ECOWAS OHZDP Workshop

Member state or organization	Workshop participant role
Republic of Benin	ECOWAS Member State
Burkina Faso	ECOWAS Member State
Republic of Cape Verde	ECOWAS Member State
Republic of Côte d'Ivoire	ECOWAS Member State
Republic of the Gambia	ECOWAS Member State
Republic of Ghana	ECOWAS Member State
Republic of Guinea	ECOWAS Member State
Republic of Guinea Bissau	ECOWAS Member State
Republic of Liberia	ECOWAS Member State
Republic of Mali	ECOWAS Member State
Republic of Niger	ECOWAS Member State
Federal Republic of Nigeria	ECOWAS Member State
Republic of Senegal	ECOWAS Member State
Republic of Sierra Leone	ECOWAS Member State
Republic of Togo	ECOWAS Member State
Economic Community of West African States, Regional Animal Health Centre (RAHC)	Observer
Inter-State School for Sciences and Veterinary Medicine (EISMV)	Observer
National Laboratory for Veterinary Studies and Research (LNERV)	Observer
Nigeria Centre for Disease Control (NCDC)	Observer
United States Agency for International Development (USAID)	Observer
United States Defense Threat Reduction Agency (DTRA)	Observer
United States Department of Agriculture (USDA)	Observer
West Africa Livestock and Innovation Center (WALIC)	Observer
World Health Organization (WHO)	Observer
Food and Agriculture Organization (FAO)	Observer and Facilitator
United States Centers for Disease Control and Prevention (CDC)	Observer and Facilitator

### During the workshop

The ECOWAS OHZDP Workshop was hosted in Dakar, Senegal from December 3–7, 2018. A typical OHZDP workshop is two days when conducted at a national level, however the CPT agreed that to adequately address the needs of the 15 Member States the length of the workshop should be extended to five days. Facilitators adapted the typical agenda to incorporate presentations from each Member State, expanded time for each section of the OHZDP Process for ensuring in-depth discussions and consensual deliberations, and added time for a final in-person review of the workshop report. This workshop also incorporated simultaneous interpretation for English, French, and Portuguese to accommodate the workshop participants from the region.

On the first day of the workshop, an opening ceremony was held at the Prime Minister of Senegal’s Office where each Member State provided an overview of the One Health infrastructure in their country.

Once the workshop began, participants reviewed and finalized the initial zoonotic disease list for prioritization, developed five criteria for ranking the zoonotic diseases, and developed one categorical question for each criterion through small and large group discussions. All questions had ordinal, binomial, or multinomial answers. Participants determined a score for each answer choice that would be used to assess each zoonotic disease under consideration. Each Member State then ranked their preferences for the relative importance of each criterion. A facilitator entered each country’s ranking into the OHZDP Tool to calculate a weight for each criterion that reflected the overall groups ranking; the criterion weights are calculated using the Analytical Hierarchy Process previously described [6].

Usually the OHZDP Tool is limited to 12 spaces for voting members, however since 15 countries were involved in this workshop, the CPT discussed the importance of each country receiving their own individual input and CDC facilitators adapted the OHZDP Tool to accommodate input from the 15 Member States.

Next, facilitators and participants scored each of the zoonotic diseases by answering the question developed for each criterion. The data used to answer each question were identified through an extensive literature search prepared in advance of the workshop, as well as information from WHO, the World Organisation for Animal Health (OIE), ProMED, and other relevant websites. The literature search incorporated both peer-reviewed and grey literature. Search terms included the zoonotic diseases on the initial list, all countries in the region, ECOWAS, and disease transmission, severity, pandemic and epidemic potential, economic impact, prevention and control, or environmental impact. Data for these thematic areas were collected in Microsoft Excel™ for each zoonotic disease at the region and country level. If information for a zoonotic disease was not available for the region, global data or subject matter experts were consulted.

After scoring each of the zoonotic diseases, the ranked zoonotic disease list was determined using a decision tree analysis built into the OHZDP Tool. Each weighted criterion was applied across each question’s answers for each zoonotic disease. The scores of all five questions for each zoonotic disease were summed. The highest raw score was then normalized, giving that zoonotic disease a normalized score of one.

The zoonotic diseases with their raw and normalized scores were presented to the workshop participants for discussion. Workshop participants used the ranked OHZDP list as a starting point to for discussions during which a final priority zoonotic disease list was selected. Participants reviewed the ranked OHZDP list and then began discussions by considering the top ten ranked zoonotic diseases produced from the OHZDP Tool. After selecting six zoonoses for their final list from the top ten ranked zoonotic diseases from the OHZDP Tool, participants chose to select additional diseases from the list based on regional priorities. Participants chose to merge viral hemorrhagic fevers into one category which included a couple of these zoonoses that were ranked lower by the tool, but still important to the region from a One Health perspective. The final list of prioritized disease was agreed to by consensus of all ECOWAS voting members. After the Member States confirmed the priority zoonotic diseases, participants collaboratively developed recommendations for next steps and action plans to address the priority zoonotic diseases using a One Health approach for ECOWAS.

ECOWAS Member States finalized the priority zoonotic disease list, recommendations for next steps from the OHZDP Workshop for ECOWAS, and the workshop report as a group on the last day of the workshop.

### Analysis of data

For the breakdown of participants, Member State representatives that attended the workshop were counted once as representing human health, agriculture, or wildlife/environment using Microsoft Excel™. Representatives from human health or public health ministries were counted as “human health”, representatives from livestock or agricultural ministries were counted as “agriculture”, and representatives from wildlife or environment ministries were counted as “wildlife/environment”.

### Ethical considerations

The primary intent of this workshop was for public health practice and did not conduct human subjects research. Individual participant level data was aggregated and analyzed anonymously thereby maintaining confidentiality.

## Results

### Participants

There were 16 representatives on the CPT. The ECOWAS OHZDP Workshop had a total of 66 participants, including facilitators, Member States, and observers (Table 1). Ten facilitators from CDC and FAO facilitated this regional workshop and 56 participants were from Member States and observers. Table 1 shows the list of participating organizations for this workshop.

Of the 15 ECOWAS Member States, ten (67%) countries had representation from human, agriculture, and environmental health sectors, four (27%) had representation from two of the sectors, and one (7%) had representation from one sector. Thirty-nine (71%) participants were from ECOWAS Member States; eleven (28%) represented human health, 14 (36%) represented agriculture, and 14 (36%) represented wildlife/environmental ministries. Sixteen (29%) participants were observers representing ECOWAS, U.S. government organizations, United Nations organizations, and research institutes.

### Initial zoonotic disease list for prioritization

Member States agreed upon an initial list of 30 zoonotic diseases for prioritization (Table 2). Of the initial zoonotic disease list, 12 (40%) were viruses, 10 (33%) were bacteria, and 8 (27%) were parasites.

**Table 2:** Final ranked zoonotic diseases, raw scores, and normalized final scores

Rank	Zoonotic disease	Raw score	Normalized final score
**1**	**Anthrax**	**1**	**1**
2	Leishmaniasis	0.920634909	0.920634909
3	Echinococcosis	0.841269818	0.841269818
**4**	**Rabies**	**0.832673256**	**0.832673256**
**5**	**Rift Valley fever**	**0.824734211**	**0.824734211**
**6**	**Zoonotic influenzas**	**0.824734211**	**0.824734211**
**7**	**Trypanosomiasis**	**0.824734211**	**0.824734211**
8	Leptospirosis	0.783398542	0.783398542
**9**	**Ebola**	**0.766862935**	**0.766862935**
10	Brucellosis	0.741733628	0.741733628
**11**	**Yellow fever**	**0.687497844**	**0.687497844**
12	Campylobacteriosis	0.662368537	0.662368537
**13**	**Crimean-Congo hemorrhagic fever**	**0.649468422**	**0.649468422**
14	West Nile Fever	0.649468422	0.649468422
**15**	**Zoonotic tuberculosis**	**0.645832930**	**0.645832930**
16	Listeriosis	0.607475235	0.607475235
17	Plague	0.607475235	0.607475235
18	Fascioliasis	0.607475235	0.607475235
19	Cysticercosis	0.566467839	0.566467839
20	Salmonellosis	0.562832346	0.562832346
21	Escherichia coli	0.416999417	0.416999417
**22**	**Marburg**	**0.415673839**	**0.415673839**
23	Middle East Respiratory Syndrome (MERS)	0.415673839	0.415673839
24	Monkeypox	0.412695864	0.412695864
25	Toxoplasmosis	0.409060372	0.409060372
26	Hepatitis E	0.329695281	0.329695281
**27**	**Lassa fever**	**0.313159674**	**0.313159674**
28	Q fever	0.307543948	0.307543948
29	Trichinellosis	0.291665859	0.291665859
30	Dracunculiasis	0.233794583	0.233794583

The priority zoonotic diseases selected by ECOWAS Member States are shown in bold

### Criteria and questions

The five agreed upon criteria and their associated weights for the ECOWAS region were severity of disease (36%), prevention and control capacity (19%), epidemic or pandemic potential (18%), ability to detect (16%), and socio-economic and environmental impact (12%) (Table 3). The questions and associated answers with point values for scoring developed by the ECOWAS Member States to assess each criterion are found in Table 3.

**Table 3:** Final criteria, criterion weights and associated question used for ranking the zoonotic diseases

Criterion	Criterion weight	Question and answer choices
Severity of disease	0.36	What is the case fatality rate^a^ for the disease in animals and human in the ECOWAS region? • Case fatality rate in one or both sectors ≥ 15% – 2 points • Case fatality rate in one or both sectors ≥ 5—< 15% – 1 point • Case fatality rate in both sectors < 5% – 0 points
Prevention and control capacity	0.19	Is there any effective preventive or therapeutic measure (vaccine or antimicrobial) for humans and/or animals? • Animals and humans – 2 points • Animals or humans – –1 point • Neither – 0 points
Epidemic or pandemic potential	0.18	Has the pathogen caused an epidemic (human or animal) in the ECOWAS region? • Within the past 5 years – 2 points • Within the past ≥ 5 and < 10 years – 1 point • Never or more than 10 years ago – 0 points
Ability to detect	0.16	Is there a One Health surveillance system^b^ for this disease (investigation, laboratory, or data sharing) in the ECOWAS region? • For all three sectors – 2 points • For two sectors – 1 point • For one sector or none of the sectors – 0 points
Socio-economic and environmental impact	0.12	Is the disease on the OIE list of notifiable diseases^c^ and is the disease potentially affected by climate change (host range, vector or reservoir)? • Yes to both – 2 points • Yes to one – 1 point • No – 0 points

^a^ If data from the ECOWAS region was not available, global data was used

^b^ If five or more ECOWAS countries had a surveillance system for a disease in a particular sector, the sector was considered to have a surveillance system for that disease

^c^ The OIE list was agreed upon by the Member States as a proxy to measure socio-economic impact

### Priority zoonotic diseases for the ECOWAS Region

After scoring each zoonotic disease for each of the developed questions, a ranked list and the normalized final score for each zoonotic disease was generated from the OHZDP Tool (Table 2). ECOWAS Member States reviewed and discussed the ranked zoonotic disease list and agreed upon a list of seven priority zoonotic diseases for ECOWAS. The final priority zoonotic diseases for the ECOWAS region are anthrax, rabies, Ebola and other viral hemorrhagic fevers (for example, Marburg fever, Lassa fever, Rift Valley fever, Crimean-Congo Hemorrhagic fever), zoonotic influenzas, zoonotic tuberculosis, trypanosomiasis, and yellow fever (Table 2). Participants chose to merge viral hemorrhagic fevers into one category which included a couple of these zoonoses that were ranked lower by the tool, but still important to the region from a One Health perspective.

### Recommendations for next steps and action plans

Workshop participants discussed recommendations and further actions that could be taken, using a regional, One Health approach to address the priority zoonotic diseases and were organized into thematic areas including One Health coordination, surveillance and laboratory, preparedness and response, prevention and control, workforce development, and research needs. Participant recommendations are summarized in Table 4.

**Table 4:** Recommendations agreed upon by ECOWAS for the priority zoonotic diseases (PZDs) and One Health collaboration

Thematic areas	Proposed recommendations
One Health coordination	Establish strong regional and national One Health coordination within ECOWAS, including both ministerial and technical-level coordination
	Establish a network to facilitate communication, collaboration, and coordination among national level points of contact to address One Health issues for PZDs
	Create opportunities to socialize One Health at single sector meetings
	Establish an appropriate mechanism to share key reports, meeting outcomes, documents, and guidance across One Health focal points
	Create a governance document including roles and responsibilities, guidance for building sustainability, shared budget, and logistics for national and regional One Health coordination
	Develop a One Health strategic plan for the ECOWAS region with a focus on the priority zoonotic diseases
	Advocate and increase awareness with political leadership and funding to support One Health activities and strategic plans
	Facilitate the sharing and linkage of results of One Health assessments and tools used in countries
	Ensure One Health advocates and focal points use consistent language around One Health
	Advocate for engagement of all 15 member states in major international projects with regional One Health relevancy
Surveillance and laboratory	Strengthen and expand existing relevant regional surveillance networks to incorporate the integration of identified PZDs
	Improve the integration of human, animal, and environmental sectors to strengthen a One Health approach to surveillance
	Enhance existing surveillance platforms and incorporate the environmental sector for data sharing related to PZDs
	Develop coordinated surveillance plans and conduct joint investigations for PZDs that incorporate One Health sectors
	Identify gaps and standardize wildlife surveillance across the same ecosystems at the sub-regional level
	Create regional guidelines and standard practices for linking surveillance data and systems
	Strengthen existing laboratory and diagnostic capacity and integrate these capacities among One Health sectors
	Strengthen biosafety and biosecurity for laboratories
	Conduct subregional risk mapping of the priority zoonotic diseases to guide and focus surveillance efforts
Response and preparedness	Develop response plans for all PZDs using a One Health approach. Review and update existing plans to ensure they include all One Health sectors at the national, sub-regional, and regional level. Plans should be exercised to ensure coordination between One Health sectors and countries remains strong
	Harmonize response plans and standard operating procedures between member states for the PZDs for cross-border assistance with outbreaks
	Improve communication by sharing weekly epidemiological reports per sector to ensure all sectors can effectively collaborate and respond
	Train One Health rapid response teams to respond to outbreaks at the national or regional levels. Develop a roster of trained individuals within the region
	Expand information sharing and coordination structures created during the establishment of Emergency Operations Centers
Prevention and control	Develop and synergize prevention and control programs across One Health sectors and vaccine requisition for the priority zoonotic disease
	Develop technical networks to coordinate activities for the PZDs at the national, sub-regional, and regional levels
	Harmonize sub-regional and regional legislative efforts for the control and prevention of PZDs
Workforce development	Conduct human resource mapping to evaluate current capacity, infrastructure, training institutions, and opportunities for One Health
	Implement training and capacity building programs at the regional, subregional, or national level to build and expand the core workforce among the human health, animal health, and environmental health sectors
	Train laboratory staff on biosafety and biosecurity to build and strengthen diagnostic capacity and link between One Health sectors
	Provide risk communication training and train dedicated communications staff across sectors
	Establish a trained group of One Health professionals to respond jointly to outbreaks
	Training, field experience, and exercises should be conducted jointly through a One Health approach bringing together One Health sectors
	Identify and allocate resources to ensure that sustainable in-service training is provided to establish and maintain a strong workforce able to address priority zoonotic diseases and other One Health Issues
Research needs	Convene experts from One Health sectors across ECOWAS to identify specific research needs for PZDs
	Develop a regional network of multisectoral and multidisciplinary researchers whose work focuses on PZDs to facilitate information sharing
	Ensure the regional network of researchers is linked with other One Health networks, filed epidemiologists, laboratorians, and the regional One Health coordination mechanism

## Discussion

ECOWAS was the first region in the world to bring together representatives from all relevant sectors from each member state to utilize the OHZDP Process to prioritize zoonotic disease of greatest regional concern and recommendations for next steps and action plans for One Health collaboration [22]. The final ECOWAS regional priority zoonotic disease included seven zoonotic diseases (Table 2). At the time of this ECOWAS regional workshop, six (40%) of 15 ECOWAS Member States (Burkina Faso, Côte d’Ivoire, Ghana, Mali, Sierra Leone, and Senegal) had previously conducted an OHZDP workshop at the national level [7].

In order to effectively address the ECOWAS priority zoonotic diseases, strong regional and national One Health coordination is critical to enhance coordination, collaboration, and communication across sectors involved in addressing zoonotic diseases and related One Health concerns [15]. During the workshop, recommendations were made to establish national and regional level multisectoral, One Health coordination mechanisms. At the time of this workshop, nine (60%) of the 15 ECOWAS Member States (Burkina Faso, Cote d’Ivoire, Ghana, Guinea, Liberia, Mali, Nigeria, Sierra Leone, Senegal) had established One Health coordination mechanismsand the others were in the process of standing them up [17–19]. While countries within the region were at various levels in establishing One Health coordination mechanisms, support for resources, guidance, and engagement from all sectors is still needed [23, 24]. In addition to establishing these One Health coordination mechanisms, recommendations were made to continue information and data sharing across sectors throughout the region.

During the ECOWAS OHZDP Workshop, recommendations developed were aligned with regional and national One Health recommendations previously made. In 2020, A Framework for One Health Practice in National Public Health Institutes was published by Africa CDC [21] which included similar themes to the recommendations made in the ECOWAS OHZDP workshop. Aligning recommendations and building upon the implementation of One Heath tools is encouraged to continue advancing and strengthening One Health across ECOWAS.

Eight regional taskforces involving multiple partners to address specific priority zoonotic diseases have been established including (1) surveillance and health information systems, (2) preparedness and response, (3) risk communication, (4) research and training, (5) laboratory, (6) antimicrobial resistance, (7) environmental health and climate change, and (8) animal health. Since the workshop, key partners in West Africa, like The World Bank, German Development Agency (GIZ), German Development Bank (KfW), French Development Agency (AFD), US CDC, USAID, and others, are collaborating with the ECOWAS secretariat through the Regional Center for Surveillance and Disease Control to advance the recommendations for the priority zoonotic diseases.

Strengthening surveillance activities and regional surveillance networks for the priority zoonotic diseases was identified as an important next step. RAHC and the RCSDC have ongoing collaborative surveillance activities across the region. Existing surveillance programs within the region, like the World Bank’s Regional Disease Surveillance System Enhancement (REDISSE) project [25] and the regional DHIS-2 platform [26], have helped support national and regional surveillance for zoonotic diseases. While existing surveillance programs exist in the region, additional efforts are needed to continue strengthening surveillance activities for the priority zoonotic diseases across the region. Recommendations to develop standardized guidelines and practices for wildlife surveillance were also identified. Since the OHZDP workshop for the ECOWAS region, further efforts have been made to harmonize surveillance and reporting for the priority zoonotic diseases across sectors.

ECOWAS Member States discussed the need to strengthen and integrate existing laboratory networks and diagnostic capacity between sectors across the region. Reference laboratories for each sector within the region were recommended to have diagnostic capacity for each of the priority zoonotic diseases. Within ECOWAS, twelve regional reference laboratories exist for human health. The human health reference laboratories in the region have diagnostic capacity for all of the priority zoonotic diseases, while two regional laboratories are designated as animal reference laboratories with diagnostic capacity for the seven priority zoonotic diseases. Improved coordination across laboratories between countries in the region, including national and regional specimen transport networks, was recognized as a priority for developing sustainable lab networks. Enhanced biosecurity and biosafety were also identified as efforts that would strengthen laboratory safety and capacity.

ECOWAS Member States recommended that response plans be developed across all relevant One Health sectors for each of the priority zoonotic diseases for the national, sub-regional, and regional levels. ECOWAS Member States also emphasized the importance of conducting exercises for developed plans to ensure One Health coordination and preparedness across the region. Integrating One Health during the establishment of Emergency Operations Centers in the region can further strengthen coordination and collaboration around health emergencies. ECOWAS plans to develop a strategic plan for One Health implementation as well as disease specific plans for the priority zoonoses.

ECOWAS Member States supported ideas to develop a roster of trained One Health professionals who can serve on One Health rapid response teams to support national or regional-level response. These teams can be built off of the existing ECOWAS Regional Rapid Response Teams [27–29]. One Health regional rapid response teams have been established in all 15 ECOWAS countries as coordinated investigation and response is encouraged for zoonotic diseases [15]. Regular regional communication and coordination should be established to address ongoing preparedness and response needs for the region.

ECOWAS Member States also identified various ways to prevent and control the priority zoonotic diseases. Zoonotic diseases that had existing effective preventative and therapeutic measures for both humans and animals were ranked higher than diseases that did not have these measures. While these measures exist globally, ensuring the availability of medical countermeasures and vaccines for both human and animal health sectors throughout the region is extremely important. Conducting assessments and developing plans to control each of the priority zoonotic diseases were also recommended. For example, assessment tools, like the Stepwise Approach for Rabies Elimination [30] and the Framework for Enhancing Anthrax Prevention and Control [31], can help identify opportunities for improvement around control measures such as surveillance, diagnostic capacity, and existing or needed policy recommendations for each of the priority zoonotic diseases within the ECOWAS region. Enhancing and synergizing surveillance activities and technical working groups across the region were also encouraged. At the policy level, legislative efforts for prevention and control activities that involve human, animal, and environmental health sectors related to the control of the priority zoonotic diseases could further be harmonized for the greatest impact.

A One Health approach should be applied to workforce development including in the medical, veterinary, and environmental health sectors. Efforts for mapping human resources and training opportunities to understand capacity and infrastructure in the region across One Health sectors were recommended. Participants also suggested that programs continue building and expanding the core One Health workforce across regional, sub-regional, and national levels. Within the region, workforce development programs, like Field Epidemiology Training Program (FETP) and Field Epidemiology and Laboratory Training Program (FELTP) [32], In Service Applied Veterinary Epidemiology Training (ISAVET) [33], and REDISSE trainings for participating countries [34], exist. Participants recommended that these workforce development programs continue to emphasize a One Health approach by training human, animal, and environmental health sectors together at sub-national, national, sub-regional, and regional levels.

Recommendations to identify and discuss research gaps across One Health sectors within the region were made; recommendations focused on the priority zoonotic diseases to address gaps in prevention, detection, and response to these threats. For example, development of a regional network of One Health researchers would be beneficial to share information and knowledge and address research gaps. During a 2019 One Health technical meeting within the ECOWAS region, recommendations were made that additional research was needed to inform practice [23]. This recommended regional research network can serve as an opportunity to address research gaps across the One Health sectors within the region. Additionally, the regional network of researchers should be linked to other One Health networks, field epidemiologists and laboratorians, and the regional One Health coordination mechanism.

ECOWAS was the first region in the world to utilize the OHZDP Process to develop a regional priority zoonotic disease list and recommendations. The OHZDP Process has been conducted in over 26 locations at the subnational, national, and regional levels; these past subnational and national workshops and this regional ECOWAS workshop highlight the scalability of the OHZDP Process [7]. Additional regions around the world can utilize the OHZDP Process to establish or continue strengthening regional One Health collaborative efforts.

## Conclusions

The unique regional prioritization brought together One Health sectors from 15 ECOWAS Member States to prioritize the zoonotic diseases that posed the greatest threat to the region. To address the developed recommendations to mitigate zoonotic disease threats in the region using a One Health approach, additional resources and policies are needed. This regional prioritization provides a framework of collaboration between the ECOWAS secretariat, ECOWAS Member States and other partners and has triggered collaborative and innovative interventions for tackling zoonotic diseases in West Africa. Other regions across the world that are aiming at strengthening One Health can also benefit from conducting an OHZDP and this collective approach. The effective mitigation of the impact of zoonotic diseases in the ECOWAS region requires strong One Health collaboration and partnerships. Strengthening national and regional One Health coordination mechanisms and building surveillance, laboratory, preparedness, response, workforce, and research capacity will allow ECOWAS Member States to advance One Health, most effectively address the region’s priority zoonotic diseases, and have the biggest impact on improving health outcomes for both people and animals living in a shared environment.

## Data Availability

A final workshop report for the One Health Zoonotic Disease Prioritization for ECOWAS can be found at www.cdc.gov/onehealth/what-we-do/zoonotic-disease-prioritization/completed-workshops.html. Additional information may be made available to researchers who submit a proposal to the corresponding author.

## Abbreviations

AFD: Agence française de développement
CDC: U.S. Centers for Disease Control and Prevention
ECOWAS: Economic Community of West African States
FAO: Food and Agriculture Organization of the United Nations
FETP: Field Epidemiology Training Program
FELTP: Field Epidemiology and Laboratory Training Program
GIZ: Deutsche Gesellschaft für Internationale Zusammenarbeit
ISAVET: In Service Applied Veterinary Epidemiology Training
OHZDP: One Health Zoonotic Disease Prioritization
OIE: World Organisation for Animal Health
RAHC: Regional Animal Health Center
RCSDC: Regional Center for Surveillance and Disease Control
REDISSE: Regional Disease Surveillance System Enhancement
SARE: Stepwise Approach for Rabies Elimination
UNEP: United Nations Environnent Programme
USAID: United States Agency for International Development
WHO: World Health Organization
